# Microwave Fill Level Inspection System for Industrial Packaged Products

**DOI:** 10.3390/s25247578

**Published:** 2025-12-13

**Authors:** Calin I. Maraloiu, Jorge A. Tobón Vasquez, Marco Ricci, Francesca Vipiana

**Affiliations:** 1 Department of Electronics and Telecommunications, Politecnico di Torino, 10129 Torino, Italy; calin.maraloiu@polito.it; 2 Wavision S.r.l., 10129 Torino, Italy; jorge.tobon@wavision.it (J.A.T.V.); marco.ricci@wavision.it (M.R.)

**Keywords:** microwave antennas, microwave propagation, microwave sensors, vivaldi antennas

## Abstract

Fill level control is one of the strict checks required when inspecting industrially packaged products. The purpose is both to ensure the content conformity according to the declared label information and to preserve the reliability of brand trust, strongly influenced by the customer’s evenness perception of the marketed items. To this aim, choosing the right technology is not an easy task: content and packaging material properties are essential to establish the suitability of a product to the fill level machine type. In this paper, we propose a novel approach, based on microwaves, to address this issue. The designed microwave inspection system consists of two Vivaldi antennas working between 1 and 18 GHz. We show its applicability to water, oil and alcohol-based products moving on conveyor belts at production speed. The performed experiments demonstrate good accuracy and efficiency of level classification and fault rejection in real-time processing.

## 1. Introduction

Inline fill level inspection of packaged items is a critical verification process that manufacturers perform on production lines to comply with industrial standards and legal regulations concerning the amount of product delivered to the customer. Checkweighers have limited applicability because of their bulkiness, low throughput, and frequently required routine servicing procedures to prevent calibration loss, wear and tear, and sensitivity to humidity and temperature of the load cell [1]. Therefore, modern facility plants have been focusing on fill-level control technologies. To state the correct amount of content, the measured level is compared against a target height level. These technologies include gamma rays, high-frequency (HF) capacitive, infrared (IR), x-rays and optical camera fill level devices [2]. Gamma ray machines are nowadays on the edge of cutoff: the increasingly stricter safety regulation associated with their usage deletes any future perspective. HF-capacitive fill level control devices are able to perform the height check by converting the variation in the liquid volume into a capacitance difference through a resonant circuit. However, they have issues assessing viscous, non-aqueous products or high alcohol content liquids. Moreover, they cannot be applied if metallic parts (caps or films) are present in the inspection region [3,4]. IR systems measure the intensity level of the received signal only if the package of the product under test is transparent to the IR radiation and the product is not foiled or labeled. X-rays are probably the most commonly adopted fill-level inspection machines in production lines. However, such units entail high energetic consumption and require specific radiation protection and training protocols. Also, a limitation is posed by the small inspected region: in some cases, to detect both over-fill and under-fill conditions, a combination of more sensor pairs is required, increasing complexity and costs. Finally, optical camera fill inspection systems have been deployed for fill sensing. These systems require a certain degree of translucency, transparency, and purity of the materials. Moreover, there are problems if droplets, caused by the trickling fill process, are present [5,6,7].

In this paper, we propose a microwave (MW) fill level control system as a novel inspection approach. MW systems exploit the electromagnetic (EM) radiation, sourced and sensed by antennas working in the MW range, to retrieve useful information for various applications. Up to now, the industrial applications exploit MW units mainly for static tank fill sensing applications [8,9,10,11], imaging and classification for quality control in the industrial context [12,13,14], and grain inspection [15,16]. Here, instead, we design and experimentally test an MW device for inline fill level inspection of items moving along a production line. We employ microwaves in the range of 1–18 GHz and use two Vivaldi printed antennas to verify the fill level. As the volume difference alters the EM transmitted signals, we analyze the behavior of the transmission coefficients, so that each sensed height level is mapped to a certain transmission level.

In [17], microwaves are also used for inline fill level inspection, but assuming a monotonic behavior of the fill level with respect to the amplitude of the measured transmission coefficient. This assumption is not considered in our approach because we verified that it is not always present, in particular in the case of low-loss liquids such as oil. Moreover, in [17], only one single-frequency acquisition is considered, while here multiple spatial and spectral measurements are performed. This boosts the accuracy and lifts the monotonicity restriction, allowing the extension of the industrial reach and ensuring the feasibility for different materials.

Table 1 presents a detailed comparison of the key operational capabilities of the fill-level sensing technologies currently adopted in industrial inspection systems. The analysis includes their suitability for performing fill-level inspection on moving products within production lines, as well as the use of harmless non-ionizing radiation for the sensing process. The ability to operate with containers of different opacity and to assess labeled products is also detailed. Moreover, the table considers systems compatibility with metallic containers and their capability to inspect products provided with metallic caps, which often represent challenging conditions for most conventional fill-level sensing approaches. Further performance indicators include compatibility with water-based, oil-based, and alcohol-based products, addressing a wide range of industrial liquid categories. Power consumption is additionally considered as a key metric for continuous industrial operation. Finally, the capability to perform multi-level-fill classification, rather than a simple binary decision between compliant and faulty products, is analyzed, highlighting the potential for enhanced process control of the proposed MW system.

The paper is organized as follows. Section 2 presents the architecture of the system, while Section 3 and Section 4 illustrate the classification and fault rejection methodologies and outcomes, respectively. Finally, Section 5 reports the conclusion and perspectives.

## 2. Inspection System Architecture

**Figure 1:**
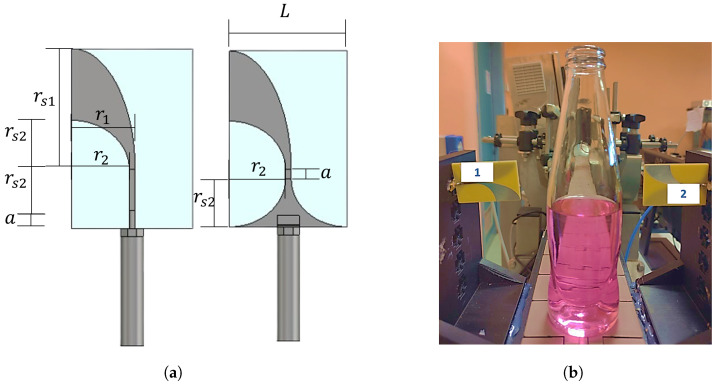
Proposed MW fill level inspection system. (**a**) Front and back sides of the Vivaldi antenna exploited as transmitter and receiver. (**b**) The two Vivaldi antennas, facing each other, mounted on a plastic support, fitting a prototype production line, and scanning the fill level of the glass bottle moving on the conveyor belt.

As shown in Figure 1, the designed MW inspection system consists of a pair of printed microstrip Vivaldi antennas, which provide and sample the EM waves necessary to cover the inspection area of the analyzed product. Vivaldi printed antennas have been successfully used in other MW sensing applications, as in the medical field [18,19,20,21].

We exploit this antenna due to its end-fire behavior: the maximum radiation is focused in line with the antenna, and this allows us to point the sensors towards the fill level, minimizing the coupling with external RF signals and the interaction with other surrounding materials. Additionally, these Vivaldi antennas show a wide-band behavior; thus, they can be deployed to perform a multi-frequency scan. The geometrical parameters of the designed antenna, reported in Figure 1, are a=2.5 mm, L=30.7 mm, $r_{1}=16.1$ mm, $r_{2}=14.6$ mm, $r_{s1}=30.7$ mm and $r_{s2}=12.5$ mm. The dielectric substrate is FR-4 with a thickness of 1.55 mm and the copper metallic layers have a thickness of 0.035 mm.

The two Vivaldi antennas are connected to a Keysight M9804A PXI vector network analyzer (VNA; Keysight Technologies, Santa Rosa, CA, USA) [22], through coaxial cables. The whole setup is mounted along a prototype production line. The speed of the conveyor belt is 20 m/min, close to those of manufacturing plants. The conveyor belt is provided with industrial centering bars that allow the bottles undergoing inspection to be aligned mechanically and minimize lateral misalignments.

**Figure 2:**
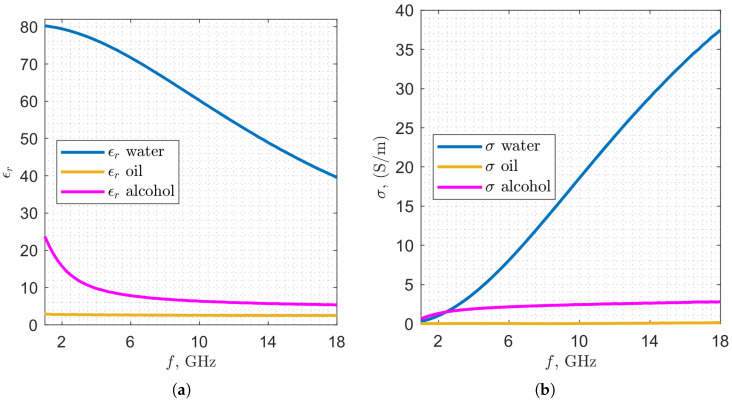
Measured values of the complex dielectric permittivity of water, oil and alcohol: (**a**) relative permittivity $\epsilon_{r}$, (**b**) electrical conductivity σ.

The liquids under trial are characterized by different dielectric properties that, together, represent the majority of beverage and food product categories. Figure 2 reports the values of the relative permittivity $\epsilon_{r}$ and electrical conductivity σ, measured through a probe [23] connected to the VNA, in the frequency range 1–18 GHz. In all the considered bandwidth, the oil exhibits low relative permittivity and low losses, while the water and alcohol have higher relative permittivity and losses (in particular water), and display more pronounced frequency-dependent behavior. Within the selected frequency range, the maximum wavelength (i.e., minimum frequency) has to provide sufficient spatial resolution to detect variations in fill level, while the minimum wavelength (i.e., maximum frequency) is constrained by the upper limit of the VNA (18 GHz). Therefore, the setup of the acquisition process is performed as follows: frequency range from 1 to 18 GHz with 69 points (0.25 GHz of step) in frequency and intermediate filter (IF) frequency set at 100 kHz, with 0 dBm input power. A standard VNA calibration process is put into place to compensate for the cable loss and phase shift, but no further preprocessing is applied on the transmission coefficient. For the spatial sampling process, a photocell is exploited to trigger the proper synchronization. We set the VNA to allow 30 spatial samples, separated from each other by a 0.2 cm step, covering an overall displacement window of 6 cm, centered with respect to the antenna position along the belt. Taking several measurements during the product movement on the belt is helpful because it allows screening the item at different points and improves accuracy due to the augmented classification information. Finally, the measured data are collected and analyzed on a laptop with an AMD Ryzen 5 5625U @ 2.30 GHz processor, 16 GB of RAM, and a 64-bit operating system.

The measurements acquisition strategy consists of a collection of transmission parameters, organized in a matrix with dimensions X×F, where X=30 is the total number of spatial samples, while F=69 is the total number of frequency points.

The selection of the spatial and spectral discretization steps is a trade-off between available information and complexity. A finer discretization step would allow more available classification information, but at the burden of the acquisition and processing time. This corresponds to the time required to measure and assess the product, and has to be compatible with the throughput of the conveyor belt to avoid slowing down the production rate. The synchronization process adopted ensures optimal throughput on the prototype system used in our laboratory. In the configuration under analysis, each acquisition trigger results in a sweep time of approximately 1.84 ms for a single antenna. Since the system employs two antennas, and considering that sampling occurs every 0.2 cm when the conveyor moves at 20 m/min, the acquisition time is constrained by the corresponding displacement interval of 6 ms. The time required to store and retrieve the acquired data in the processing unit’s RAM is negligible, if compared to this limit. For the full acquisition process, 30 spatial samples correspond to a total bottle displacement time of 180 ms.

**Figure 3:**
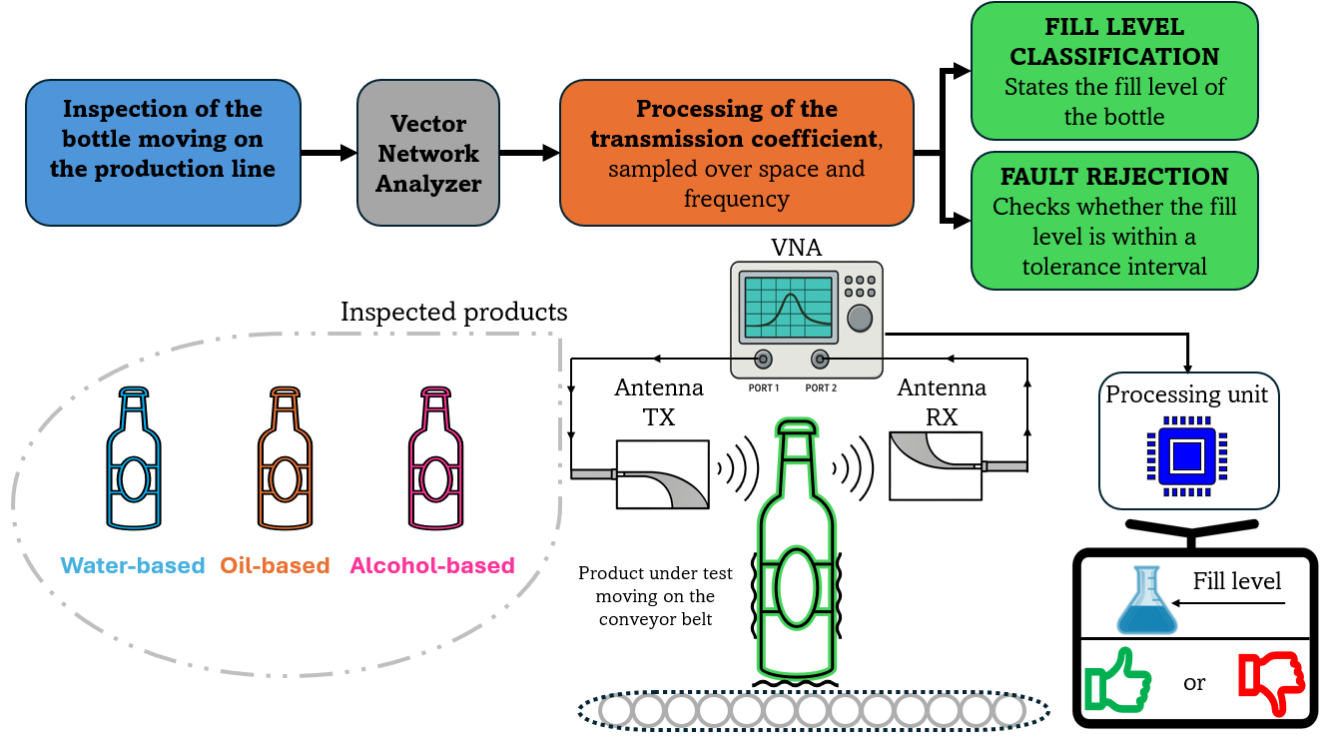
Purpose of the fill-level inspection system: classification and fault rejection.

The proposed fill-level inspection system, as detailed in Figure 3, is exploited for the following:

- classification of the fill level of an unknown test product (detailed in Section 3);
- detection of non-compliant test products to a specific target fill (detailed in Section 4).

The purpose of both applications is to ensure a real-time verification process of the packaged items, moving on the conveyor belt at a speed fit for production lines. In this analysis, the height portion of the product under test is discretized into L=31 levels. Taking reference from the literature and industrial datasheets of the commonly exploited technologies [2], the aimed accuracy is of the order of ±1.0 to ±2.5 mm in height. The product here inspected is a common 180 ml soda-lime glass transparent bottle, as shown in Figure 1. It has a base diameter of 52 mm and a height of 190 mm, with a curved shape profile. Visual opacity of the container or media does not pose a problem for microwave devices; however, a transparent item is convenient in an experimental stage. To perform accurately the fill steps, we use a weight scale with a 0.1 g tolerance [24]. It is not straightforward to carry out millimeter fill steps, since there is a non-uniform increase in the height steps. Anyhow, for the sake of completeness, the fill-height steps (in mm) associated with the liquid additions (in g) are also reported in the following sections.

## 3. Fill Level Classification

The microwave fill level control system is first exploited to classify the content amount in the fill range from 130 to 160 g for water, 120 to 150 g for oil and 100 to 130 g for alcohol (1.0 g step each).

**Figure 4:**
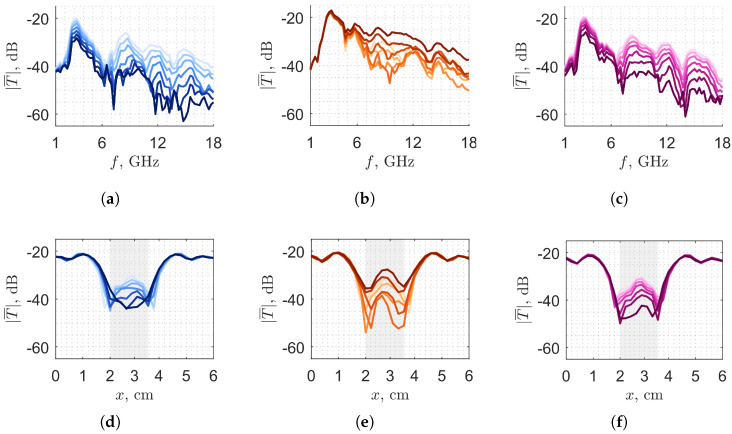
Magnitude of the averaged transmission coefficient of (**a**) water, (**b**) oil and (**c**) alcohol versus frequency at x=3cm and of (**d**) water, (**e**) oil and (**f**) alcohol versus space at f=9.25GHz. The colors, darker to lighter, map from higher to lower the volume content of the bottle in the fill range (step of 5 g). The gray regions represent the chosen trigger window.

Figure 4 shows the averaged magnitude of the measured transmission coefficients $\bar{T}$ (each data point is the average over 20 measurements), for 7 different levels (5.0 g steps). It is evident that the fill level has an impact on the pattern of the transmission coefficients, and we exploit this feature for classification. Additionally, for water and alcohol, in some frequency intervals there is a monotonic behavior with respect to the level: the higher the level, the lower the signal due to the absorption of the medium. Instead, this is not the case for oil, as it is visible in the second column of Figure 4. Furthermore, we notice that, at the beginning of the frequency bandwidth (where the wavelength is the longest, resulting in the worst spatial resolution), the impact of the different fill levels on the transmission coefficients is limited (in particular for the oil case, where the relative permittivity is low, the curves overlap); then, in the rest of the bandwidth, the transmission coefficients are clearly affected by the different fill levels for all the considered liquids.

Given the conveyor belt running at production speed, to build the classification dataset, for each level *l* belonging to the *L* fill level set, we acquire 20 [T] transmission matrices (each one of size X×F), sampled both in space *x* and in frequency *f*. Provided the set of L=31 fill level matrices, for each *l* level we have a collection of transmission coefficients $T_{x,f}$. Then, we compute the average, over the 20 classification samples, of each entry of the transmission matrices for each level *l* of the dataset, calling the respective averaged transmission coefficients matrix $\left[\bar{T}\left(l\right)\right]$ (with size X×F). The experiment is performed with arbitrary orientation of the tested bottles so that possible different glass thicknesses are accounted for.

To validate the system in real-time, we acquire 15 samples for each fill level to build a test dataset. We denote each test transmission coefficients matrix $\left[T^{\mathrm{test}}\right]$, with size X×F. For the classification procedure, we exploit a nearest-neighbor-based algorithm, mapping the unknown level test transmission coefficients set to the closest average characteristic one present in the classification dataset. Given the measured $\left[T^{\mathrm{test}}\right]$, we estimate the corresponding fill level $l_{\mathrm{est}}$ as follows:

(1) $$l_{\mathrm{est}}=\arg\min_{l}D(l)=\arg\min_{l}\sum_{x\in \tilde{X}}\sum_{f\in \tilde{F}}\left|T_{x,f}^{\mathrm{test}}-{\bar{T}}_{x,f}\left(l\right)\right|$$

that consists of retrieving the overall differences D(l), computed by summing (over the space *x* and the frequency *f* points) the modulus of the difference between the test matrix element $T_{x,f}^{\mathrm{test}}$ and each average level matrix entry ${\bar{T}}_{x,f}\left(l\right)$. In Equation (1), $\tilde{X}$ and $\tilde{F}$ are subsets of *X* and *F*, and they account for the fact that not all the $T_{x,f}$ meaningfully contribute to the classification procedure, as shown in Figure 4. In this assessment, we select the central spatial range, corresponding to 1.6 cm almost centered within the displacement window, with indices $\tilde{X}=\left[11,\ldots ,19\right]$, while keeping the whole spectral set, i.e., $\tilde{F}=\left[1,\ldots ,69\right]$. We call this selection “trigger window”, since it matches the space portion in which the bottle is moving in front of the antennas. The argument of the minimum of D(l), $l_{\mathrm{est}}$, corresponds to the classification level of the tested product. If more than one minimum is found, the resulting $l_{\mathrm{est}}$ is an average of the estimated minima.

To classify one bottle moving at 20 m/min on the conveyor belt, the processing time is approximately 4.70 ms, guaranteeing an outcome before the next bottle arrival. The data acquisition time for each trigger takes around 1.84 ms.

**Figure 5:**
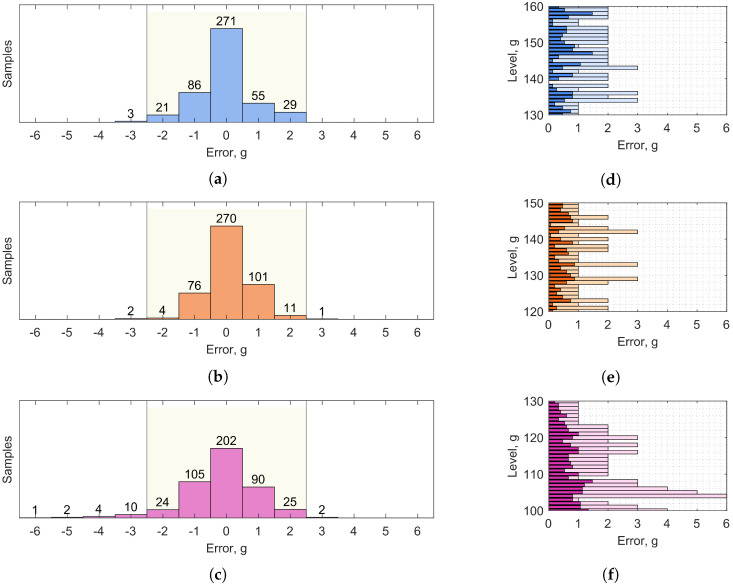
Histograms representing classification errors. Distribution error of the 465 test samples of (**a**) water, (**b**) oil and (**c**) alcohol. Average (darker color) and maximum (lighter color) errors for each level of (**d**) water, (**e**) oil and (**f**) alcohol.

Figure 5 shows, on the left, the classification distribution error in the fill level estimation for each liquid (each one constituted of 465 test samples, 15 for each level). Instead, on the right, it reports the average and maximum classification errors for each level. We can observe that the classification strategy is efficient in sorting the test dataset samples within an average error of 1.0 g for most cases. We expect that the error is mainly due to the oscillations of the liquid inside the container in motion. Oil is the most viscous among the analyzed liquids, and its surface tends to move less, followed by water and alcohol in this order. The stated behavior shows off in the lower exhibited variance in the classification and error distributions. Table 2 summarizes the hit rates, defined as the percentage between the correctly classified samples and the total tested samples, for the three considered contents, for all the fill levels, and within a classification tolerance of 0.5, 1.5 and 2.5 g, respectively. It also provides the average height fill range relative to the corresponding mass one.

## 4. Fault Rejection

The fault rejection implementation is a simplified and optimized version of the classifier described in Section 3. We start considering one target level, called in the following M, that is the level against which pass or rejection is determined. For comparison simplicity, we choose a common height level for all the materials, aligned with the midpoint of the antennas’ shorter edge. It corresponds to a target level of 146 g for water, 136 g for oil and 114 g for alcohol. Then, for the implemented fault rejection algorithm, we need to define the lower and upper levels with respect to the target one, and we choose 5.0 g below and above M. We label these levels L and H, respectively. This choice ensures a rejection range of 2.5 g around the target level M. The D(l) in Equation (1) is reduced to three differences that correspond to the measured transmission coefficients under test minus the lower level ones ($D_{\mathrm{TL}}$), the target level ones ($D_{\mathrm{TM}}$) and the upper level ones ($D_{\mathrm{TH}}$).

These differences form convex curves with respect to the tested fill level T. The minima of $D_{\mathrm{TL}}$, $D_{\mathrm{TM}}$ and $D_{\mathrm{TH}}$ are expected when the tested fill level corresponds to the L, M or H fill level, respectively. As T shifts away from the point of minimum of its corresponding *D*, the curve increases globally, resulting in a V-shaped profile. The reason for this is that the tested transmission pattern changes progressively from the one exhibited by the L, M or H fill levels, as mentioned in Figure 4.

According to the behavior exhibited by $D_{\mathrm{TL}}$, $D_{\mathrm{TM}}$ and $D_{\mathrm{TH}}$ with respect to each other, it is possible to define different intervals related to the fill state of the tested product. Table 3 links the intervals to the ordering of the convex functions and to the classes that we consider here. The outer intervals are associated with the cases when the bottle is either under- or over-filled, and we label these classes with −1 and 1, respectively. If the tested item aligns with the target item (T=M), then it is conforming. However, there are two other cases where the tested product is compliant: these occur when it is filled within the established admitted tolerance range (±2.5 g). These three intervals in between are mapped to the class labeled with 0.

**Figure 6:**
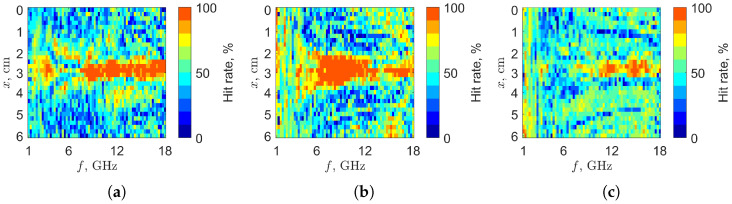
Percentage hit rate (within ±0.5 g) for the chosen target level performed on the classification dataset (20 measurements). The matrices size is 30×69, where rows correspond to triggers and columns to frequencies. (**a**) Water (target 146 g), (**b**) oil (target 136 g) and (**c**) alcohol (target 114 g) percentage hit rates.

**Figure 7:**
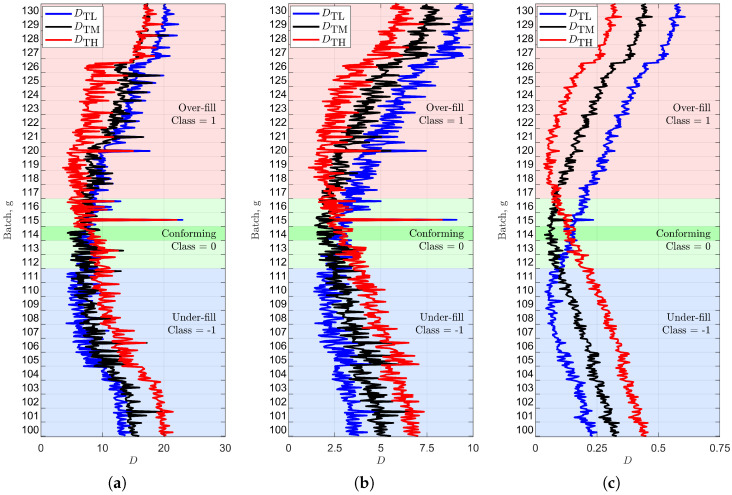
Rejection for the chosen alcohol target level according to the proposed classification parameter selection: (**a**) considering all parameters, (**b**) considering only trigger window parameters, and (**c**) with optimized filtering selection applied. The dark-green region corresponds to the target batch (114 g), light-green regions to the classification tolerance (±2.5 g for the conforming batches), while over-fill and under-fill batches are marked with red and blue areas, respectively.

The classification and test datasets employed for this task are the same ones discussed in Section 3. We also perform an optimization procedure, for the considered target level, by re-classifying the classification dataset against the average matrix $\left[\bar{T}\left(l\right)\right]$ derived from it, in order to select the most relevant classification parameters $T_{x,f}$. This is reported in Figure 6 in terms of the hit rate of each $T_{x,f}$. As expected from Figure 4, the central parameters with respect to the vertical axis correspond to the most relevant ones (red pattern). In Figure 7, we report, for the alcohol case (that from Figure 6 seems the most challenging), $D_{\mathrm{TL}}$, $D_{\mathrm{TM}}$ and $D_{\mathrm{TH}}$ with respect to the test measurements, for the different fill levels. For easier reading, we box the measurements into 31 batches (from 100 to 130 g) corresponding to the *L* considered levels: each batch (vertical step in the plot) contains 15 test measurements. Hence, according to the adopted representation, this ideally translates into V-shaped staircases. However, due to the measurement variability and noise, the resulting behavior is smoothed.

The V-shaped behavior aligns with expectations: $D_{\mathrm{TM}}$ attains its minimum when the analyzed test measurements belong to the test batch associated with the target level M. The $D_{\mathrm{TM}}$ increases as it moves away from the target level. The same behavior is obtained for $D_{\mathrm{TL}}$ and $D_{\mathrm{TH}}$: in these cases the minima are just shifted towards the batches matching, respectively, the L and H levels. In the three plots of Figure 7, the V-shaped curves are computed as follows:

The ones on the left are evaluated accounting for all the spatial samples and all the frequency points. For the central ones, the spatial samples are limited to the trigger window. Finally, the right ones are obtained by selecting the spatial samples and the frequencies so that the hit rate, computed over the classification dataset, is greater than 90%, corresponding to the red elements in Figure 6. Figure 7 (right) shows that performing a filtering process of the parameters enhances the selection accuracy, since the three curves become more distinguishable among them.

**Figure 8:**
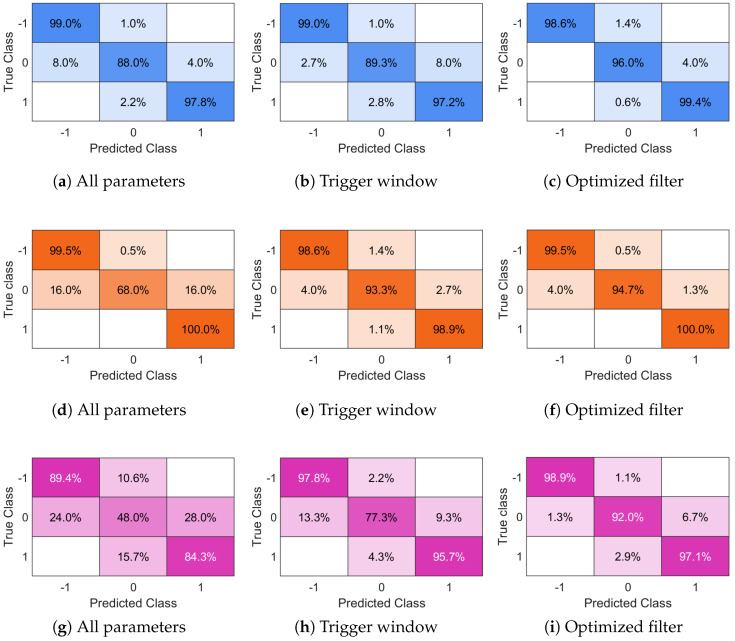
Confusion matrices for the chosen target level according to the proposed classification parameter selection. The rows correspond to the considered liquids: water (top), oil (middle), alcohol (bottom). The columns correspond to the classification parameter selection: (**a**,**d**,**g**) all parameters; (**b**,**e**,**h**) trigger window; (**c**,**f**,**i**) optimized filtering selection. The labels −1, 0, and 1 indicate under-fill, conforming, and over-fill classes, respectively.

Finally, we verify the performance of the fault rejection algorithm, illustrating the confusion matrices in Figure 8, obtained by assessing the test dataset. Keeping the most relevant $T_{x,f}$ (right column of Figure 8), as expected, improves the rejection accuracy, leading to a hit rate higher than 90%, within an acceptance range of ±2.5 g for all three considered liquids. Moreover, observing the behavior of the V-shaped curves against the expected classes (colored regions in Figure 7, labeled as shown in Table 3), it is possible to observe that the misclassifications arise from test items having T≈1/2(M+L) or T≈1/2(M+H). Therefore, classification errors, which are low for all the liquids, are the consequence of conforming-within-tolerance products being classified as over-filled or under-filled, and conversely. Under the given conditions, no over-filled item is misclassified as under-filled, nor any under-filled item as over-filled. This is also confirmed by the outcome of the confusion matrices.

It is important to ensure that adjacent bottles are properly spaced; otherwise, they may fall in the measurement window of the bottle currently under analysis. The optimization process can be exploited also for the purpose of reducing the spatial acquisition window in order to maximize the throughput, and this can be achieved without an accuracy compromise, as reported in Figure 6. The processing time per bottle is approximately 0.80 ms, with, again, a sweep time for each trigger of around 1.84 ms, as for the fill level classification (Section 3).

## 5. Conclusions and Perspectives

In this paper, a novel MW fill inspection system has been presented. The performed validation analysis showed classification capabilities that could meet the accuracy and efficiency industrial requirements, allowing the possibility of introducing, in the fill inspection context on production lines, microwave-based systems. The system has been designed and tested envisioning an industrial-scale implementation, which is reflected in the scenario used for the experimental trial. Its strengths include a small installation footprint and low power consumption, both of which reinforce its suitability for integration into production lines.

The next phase of this research will also be devoted to the transition from a VNA-based system to a low-cost deployable hardware implementation, as already proposed for biomedical applications in [25,26,27,28], with the aim of developing an even more compact, portable and stable system over the long term. Forthcoming investigations will also concern feasibility studies on other product categories (non-liquid or inhomogeneous media content and different packaging materials) and the validation for industrial purposes of the MW fill level inspection system.

## Figures and Tables

**Table 1:** Comparison table summarizing the features of the proposed MW fill-level inspection approach with respect to the currently available systems.

Technology	Gamma Rays	X-Rays	HF-Capacitive	IR	Optical	Proposed MW
Production line	✓	✓	✓	✓	✓	✓
Non-ionizing radiation	×	×	✓	✓	✓	✓
Container opacity	✓	✓	✓	✓	×	✓
Container labeling	✓	✓	×	×	×	✓
Metallic container	✓	✓	×	×	×	×
Metallic cap	✓	✓	×	×	✓	✓
Water suitability	✓	✓	✓	✓	✓	✓
Oil suitability	✓	✓	×	✓	✓	✓
Alcohol suitability	✓	✓	×	✓	✓	✓
Power consumption	×	×	✓	✓	✓	✓
Multi-level classification	×	×	×	×	×	✓

**Table 2:** Classification results and corresponding liquid fill.

Classification	±0.5 g	±1.5 g	±2.5 g
Water fill	±0.36 mm	±1.08 mm	±1.79 mm
Water hit rate	58.28 %	88.60 %	99.36 %
Oil fill	±0.40 mm	±1.19 mm	±1.97 mm
Oil hit rate	58.07 %	96.12 %	99.36 %
Alcohol fill	±0.49 mm	±1.46 mm	±2.44 mm
Alcohol hit rate	43.44 %	85.37 %	95.91 %

**Table 3:** Sorting behavior for the fault rejection algorithm.

Interval	Ordering	Region	Class
$T<\frac{1}{2}\left(M+L\right)$	$D_{\mathrm{TL}}<D_{\mathrm{TM}}<D_{\mathrm{TH}}$	Under-fill	−1
$\frac{1}{2}\left(M+L\right)\leq T<M$	$D_{\mathrm{TM}}\leq D_{\mathrm{TL}}<D_{\mathrm{TH}}$	Conforming within tolerance	0
T=M	$D_{\mathrm{TM}}<D_{\mathrm{TL}}\approx D_{\mathrm{TH}}$	Conforming to target	0
$M<T\leq \frac{1}{2}\left(M+H\right)$	$D_{\mathrm{TM}}\leq D_{\mathrm{TH}}<D_{\mathrm{TL}}$	Conforming within tolerance	0
$T>\frac{1}{2}\left(M+H\right)$	$D_{\mathrm{TH}}<D_{\mathrm{TM}}<D_{\mathrm{TL}}$	Over-fill	1

## Data Availability

The original contributions presented in this study are included in the article. Further inquiries can be directed to the corresponding authors.

## Abbreviations

The following abbreviations are used in this manuscript:

EM	Electromagnetic
HF	High frequency
IF	Intermediate frequency
IR	Infrared
MW	Microwave
RAM	Random-access memory
VNA	Vector network analyzer
